# Zinc Status and Autism Spectrum Disorder in Children and Adolescents: A Systematic Review

**DOI:** 10.3390/nu15163663

**Published:** 2023-08-21

**Authors:** Priscila Kelly da Silva Bezerra do Nascimento, David Franciole Oliveira Silva, Tássia Louise Sousa Augusto de Morais, Adriana Augusto de Rezende

**Affiliations:** 1 Postgraduate Program in Nutrition, Federal University of Rio Grande do Norte—UFRN, Natal 59056-000, Brazil; 2 Postgraduate Program in Collective Health, Federal University of Rio Grande do Norte—UFRN, Natal 59056-000, Brazil; 3 Department of Clinical and Toxicological Analyses; Federal University of Rio Grande do Norte—UFRN, Natal 59012-570, Brazil

**Keywords:** autism spectrum disorder, zinc, children, adolescents

## Abstract

Autism Spectrum Disorder (ASD) is a neurodevelopmental disorder, the prevalence of which has increased in children and adolescents over the years. Studies point to deficiency of trace elements as one of the factors involved in the etiology of the disorder, with zinc being one of the main trace elements investigated in individuals with ASD. The aim of this review is to summarize scientific evidence about the relationship between zinc status and ASD in children and adolescents. This review has been registered in the International Prospective Register of Systematic Reviews (registration number CRD42020157907). The methodological guidelines adopted were in accordance with the Preferred Reporting Items for Systematic Reviews and Meta-Analyses (PRISMA) statement. Studies were selected from an active investigation of the PubMed, Scopus, LILACS, and Google databases to search for observational studies. Fifty-two studies from twenty-two countries were included. The sample sizes ranged from 20 to 2635, and the participants ranged from 2 to 18 years old. Nine types of biological matrices were used, with hair, serum, and plasma being the most frequently used in the evaluation of zinc concentrations. Significant differences in zinc concentrations between the ASD and control groups were observed in 23 studies, of which 19 (36%) showed lower zinc concentrations in the ASD group. The classification of studies according to methodological quality resulted in high, moderate, and low quality in 10, 21, and 21 studies, respectively. In general, we did not observe a significant difference between zinc concentrations of children and adolescents with ASD compared to controls; however, studies point to an occurrence of lower concentrations of Zn in individuals with ASD. This review reveals that more prospective studies with greater methodological rigor should be conducted in order to further characterize this relation.

## 1. Introduction

Autism Spectrum Disorder (ASD) is a neurodevelopmental disorder classed among childhood mental disorders and is characterized by symptoms of deficits in communication and social interaction, and repetitive behaviors; based on the severity of symptoms and the need for support, ASD can be classified as mild, moderate, or severe [1].

There has been a significant increase in ASD prevalence, which currently affects 1 in every 160 children worldwide [2]. The etiological factors involved in the pathophysiology of ASD are still unclear. However, environmental factors, such as increased exposure to toxic metals and a consequently altered profile of trace elements, have been reported as risk factors in the development of ASD [3].

Among trace elements, zinc (Zn) is the second most abundant in the human body and participates in several metabolic processes [4], both with catalytic functions and as enzymatic, structural, and regulatory cofactors. Some of these functions are regulatory action in an inflammatory state and in oxidative stress in concert with metalloproteins [5], in addition to the elimination of toxic metals together with metallothionein (MT) [6].

In childhood and adolescence, Zn is an essential component of processes that involve growth and development [7], including the development of the nervous system [8]. Indeed, Zn deficiency at this stage causes delays in physical and mental development, including learning deficits, along with decreased immunity [9].

Overall, the evidence suggests a relationship between Zn levels and the pathophysiology of ASD. In this regard, some studies have sought to assess Zn status in individuals with ASD, as well as a complete profile of trace elements and toxic metals [10,11]. However, reviews assessing Zn status specifically in children and adolescents have not been conducted.

Given the increased prevalence of ASD, the importance of Zn in physical and mental development during childhood and adolescence, and the scarcity of systematic reviews evaluating pediatric and adolescent populations, we believe such a study would be timely and relevant. Here, we evaluated the status of Zn in children and adolescents with ASD.

## 2. Materials and Methods

This systematic review included studies published without language restrictions until July 2022. This study was based on the recommendations of the Preferred Reporting Items for Systematic Reviews and Meta-Analyses (PRISMA) [12]. The systematic review protocol was registered with PROSPERO (CRD42020157907).

### 2.1. Eligibility Criteria

The population/exposure/comparison/outcome/study design (PECOS) approach [13] was used to define the eligibility criteria. Observational studies (cross-sectional, longitudinal, case–control) with no restriction on the methods of assessment of Zn status, without language limitations, and with a population of individuals with ASD and a typical pediatric neurodevelopmental control group (2–19 years) were considered eligible for inclusion in this study. Studies in animal models that did not describe the method for assessing Zn status, systematic reviews, and studies that did not compare the ASD group and the typical neurodevelopmental control group, were excluded.

### 2.2. Search Strategy

Records were retrieved in July 2022 from the PubMed, Scopus, and Latin American and Caribbean Health Sciences Literature (LILACS) databases. Google Scholar was used to search for records in gray literature. For research carried out in gray literature, we used the methodology described by Bramer et al. (2017), with the same search strategy used in the PubMed database [14]. The search algorithm combined terms registered in the Medical Subject Headings (MeSH) platform together with the following frequently used terms in scientific articles: “zinc”, “trace elements”, “autism,” “child”, and “adolescent”, combined by Boolean operators. Supplementary Table S1 presents the search strategy used for each database.

### 2.3. Selection of Studies

After retrieving the articles from the databases, studies were selected by title and abstract by three reviewers (PKSB, DFOS, and TLSAM) independently and in pairs. At the end of the selection, the reviewers compared the selection results, and differences were resolved by a third reviewer. The studies selected based on title and abstract underwent full-text reading to assess their eligibility criteria. A manual search of the records within the references of the included articles was performed.

### 2.4. Data Extraction

The same researchers (PKSB and TLSAM) independently reviewed the full text and extracted the following data: authors, title, year of publication, magazine, country of study, n total sample, n ASD, n Control, n girls and n boys, age group, type of biological material evaluated, Zn concentration of groups and subgroups and p-value, associations between groups and subgroups, and statistical methods used. The data obtained by each of the reviewers were compared, differences were resolved by consensus, and the opinion of a fourth reviewer (AAR) was used to resolve any discrepancy. The extracted data were entered into an Excel spreadsheet form created by the authors.

### 2.5. Methodological Quality Assessment of Included Studies

Once the study selection and inclusion, and the data extraction, analysis, and synthesis, were concluded, the included studies were evaluated for methodological quality to assess the risk of bias, using the modified Newcastle–Ottawa scale (NOS) which assesses the quality non-randomized case–control and cohort studies [15].

The NOS for both case–control and cohort studies consisted of eight items. During the evaluation of a study, each item could receive 1 point (1 star), except for the *Comparability* item, where the score ranged from 0 to 2 stars. The final scores for the classification of the risk of bias were as follows: ≥6 stars, high quality; ≥4 and ≤5 stars, moderate quality; and <4 stars, low quality. Two reviewers (PKSB and TLSAM) independently performed this step. The data were compared, and the opinion of the fourth reviewer (AAR) was used to account for any discrepancy.

## 3. Results

**Figure 1:**
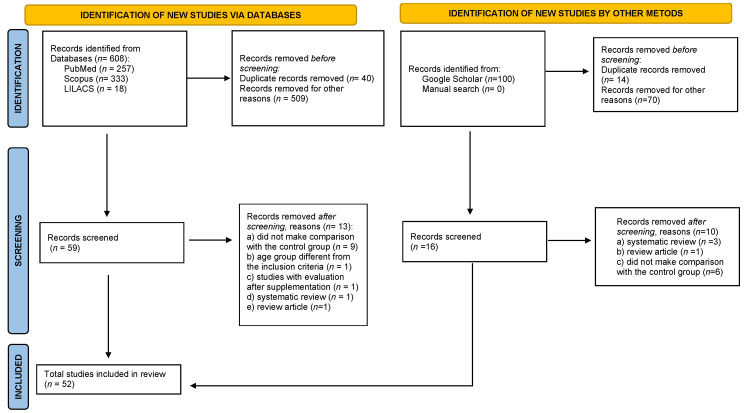
The preferred reporting items for systematic reviews and meta-analyses (PRISMA) flow chart for selection of studies.

For this systematic review of studies evaluating Zn status in children and adolescents with ASD, a total of 708 records were retrieved from the electronic databases PubMed, Scopus, LILACS, and Google Scholar gray literature. Next, the study selection by title and abstract was performed based on the eligibility criteria. A total of 129 articles were selected. When removing the duplicates, 54 articles were excluded. After reading the full-text articles, 75 studies were selected based on the eligibility criteria. After complete text analyses, 52 studies were included for data extraction, analysis, and synthesis. No articles were selected as a result of a manual search within the references of the included articles. Figure 1 shows a flowchart of the study selection.

### 3.1. Study Characteristics

Sample sizes ranged from 20 to 601, and participant ages ranged from 2 to 18 years. A total of 52 studies met the eligibility criteria, totaling 8595 participants (3969 with ASD and 4626 controls). Regarding the type of studies included, we confirmed 51 case–control studies and one cohort type. The studies were carried out in 22 countries distributed in different regions of the world, with USA, China, and Russia being the top three countries concerning the number of studies. Supplementary Figure S1 presents the percentage of studies evaluating Zn status in children and adolescents with ASD according to their country of origin.

Regarding the methodology applied in the evaluation of Zn status, nine types of biological matrices were evaluated in the studies, where hair, serum, and plasma were the most frequently used in the evaluation of Zn concentrations, in 21 (40%), 13 (25%), and 7 (13%) of the total included studies, respectively. Regarding the analytical methods for zinc quantification, the techniques used were Atomic Absorption Spectrometry—AAS (19%), Optical Emission Spectrometry with Inductively Coupled Plasma—ICP-OES (14%), Analysis by Spectrometry of Atomic Emission by Induced Plasma—ICP-AES (8%), Energy Dispersive Spectroscopy—EDS (2%), and High-Performance Liquid Chromatography—HPLC (2%), and we found that the Inductively Coupled Plasma Mass Spectrometry—ICP-MS method was the most used in studies (41%). Table 1 presents the characteristics of the included studies.

### 3.2. Methodological Quality of Studies

Regarding the assessment of the methodological quality, 10 (19%) studies were rated as high-quality (≥6 stars), 20 (39%) were rated as moderate-quality (≥ 4 ≤ 5 stars), and 21 (41%) were rated as low-quality (<4 stars). The cohort study also showed a lower methodological quality. Of the eight questions applicable to this study, four were scored. Supplementary Table S2 presents the assessment of methodological quality according to the study type.

### 3.3. Main Results of Studies

Regarding the main results observed in the total 52 included studies, significant differences between Zn concentrations of ASD and control groups were observed in 23 studies (44%): 19 (36%) showed lower Zn concentrations in the ASD group, whereas 4 (8%) showed higher Zn concentrations in this group. Supplementary Table S3 presents the studies that found differences in Zn concentrations between ASD and control groups.

In contrast, there was no significant difference in Zn concentrations between the ASD and control groups in 29 of the evaluated studies. Of these, the ASD group showed lower Zn concentrations in 16 studies (31%), and higher Zn concentrations in 10 studies (19%), compared to the control group. Of the total number of included studies, Sweetman et al. (2019), Wu et al. (2018), and Gentile et al. (1983) did not report differences in Zn concentrations between the groups [17,34,45]. Of these, Sweetman et al. (2019) reported that Zn levels exceeded the limits for reference values, where 38 children (26%) had Zn levels < 10.7 μmol/L (17 in the ASD group [23%] and 21 in the control group [29.2%]) [45].

We observed that some of the included studies analyzed Zn concentrations in relation to age, sex, associated conditions, and ASD severity. Of the studies that evaluated Zn concentrations in relation to sex, none compared Zn concentrations between female and male individuals. However, Zu et al. (2019) observed significantly lower Zn levels in 5% and 7% of males and females with ASD, respectively, but with no significant differences between the ASD and control groups for both sexes [40]. Skalny et al. (2017) observed, through correlation analyses, a significant inverse relationship between hair and serum Zn values in ASD and control female groups [27].

Additionally, of the studies that evaluated Zn concentrations in relation to sex, Wu et al. (2018) observed significantly lower Zn levels in 5% and 7% of males and females with ASD, respectively, but with no significant difference between the ASD and control groups for both sexes [40]. Skalny et al. (2017) observed, through correlation analyses, using Pearson’s coefficient I, a significant inverse relationship between hair and serum Zn values in the female ASD and control groups [27].

In addition, Zhai et al. (2019) observed significantly lower Zn concentrations between ASD and control groups for both female and male individuals [24]. However, Hawari et al. (2020) also observed higher Zn concentrations for the ASD group, although with no significant differences compared to the control group for both sexes [48].

Regarding the studies that evaluated Zn concentrations in relation to other conditions associated with ASD, Li et al. (2014) observed lower Zn concentrations in hair and serum for the ASD group with and without catatonia compared with the control group, although with no significant differences [42].

Skalny et al. (2020) observed significantly lower concentrations of Zn in the hair in a group with concomitant ASD and ADHD compared to the control group [25]. Adams et al. (2006) observed higher Zn concentrations for the ASD group with Pica syndrome compared to the ASD group without the syndrome, but with no significant differences [19].

Among the studies that evaluated Zn concentrations in relation to ASD severity, Li et al. (2014) associated the serum Zn/Cu ratio with the clinical severity of ASD (defined by the CARS score) [42]. Sehgal et al. (2019) associated Zn deficiency in whole blood with ASD in 55% of individuals with severe ASD, but with no significant difference between the groups with ASD severity degrees [41]. Sultan et al. (2019) observed significantly lower serum zinc concentrations in individuals with severe, moderate, and mild autism than in the control group, with the lowest mean observed in individuals with severe ASD [46].

Priya and Geetha (2011) observed significantly lower Zn concentrations in hair and nails between the low- and high-functioning ASD groups (mild and severe) and in relation to the control group [28]. Similarly, Zhang et al. (2021), observed significantly lower serum Zn concentrations in ASD groups for all severity levels, with the lowest results for the most severe ASD [60]. In contrast, Chehbani et al. (2020) and Wu et al. (2018) did not observe an association between Zn concentrations in plasma and erythrocytes and ASD severity [37].

## 4. Discussion

According to the studies reviewed here, the evidence does not point to a significant difference between Zn concentrations in children and adolescents with ASD compared to controls. However, most studies did observe lower Zn concentrations in individuals with ASD. Among the studies that obtained significant results in Zn concentrations between the two groups, most found lower concentrations in the ASD group than in the controls.

It is noteworthy that among the studies that observed lower Zn concentrations for individuals with ASD, most did not show a Zn deficiency when compared to Zn cutoff points for children younger than 10 years (65 µg/dL) and greater than 10 years (70 µg/dL) in plasma/serum [67]. Although the cutoff points for Zn in the hair of infants and young children have not been established, a mild Zn deficiency has been associated with values less than 70 µg/g in spring or summer and less than 110 µg/g (1.68 µmol/g) in winter. Overall, only three of the total studies revealed Zn deficiency in individuals in the ASD group [3,42,45].

Lower Zn concentrations in individuals with ASD were also observed in 2016 by Babaknejad et al., in a systematic review and meta-analysis that evaluated 11 articles published between 1978 and 2012, performed in five countries [68]. In this study, although no significant differences in Zn concentrations in the hair, nails, and teeth were found between the control and ASD groups, a significant difference was observed in plasma Zn concentrations. According to a meta-analysis conducted by Saghazadeh et al. (2017), 36 studies that evaluated not only Zn but also the profile of trace elements (boron, cobalt, chromium, copper, iron, iodine and magnesium, molybdenum, and selenium), found significantly lower Zn concentrations in whole blood for the ASD group compared to controls, in 12 of the studies [69].

Among the studies included in this review, high heterogeneity was observed concerning the types of biological matrices used in the analyses of Zn concentrations, which may likely be associated with the divergences in the findings of the included studies. Moreover, a method that is reliably more appropriate for assessing the state of this trace element becomes difficult, because the body zinc content in humans is effectively allocated in its largest part in the intracellular environment [70].

However, plasma zinc has been more recommended and used in biochemical analyses to assess the risk of zinc deficiency in populations, in addition to dietary and linear growth assessment in children and adolescents [71,72]. Nonetheless, we observed a low percentage of studies carried out with analyses in plasma (12%) and an absence of studies jointly evaluating aspects of diet and linear growth of individuals. We also observed heterogeneity in relation to the analytical methods used in the quantification of zinc; although the ICP-MS method was the most used, we did not observe a standardization regarding the method in relation to the biological matrices evaluated.

A considerable number of studies (49%) analyzed hair Zn concentrations. We found that this type of biomarker was frequently used in studies involving individuals with ASD, due to the advantages of the associated noninvasive and easy-to-collect method. Indeed, hair Zn concentrations have been considered as a potential biomarker, although there are still no established cutoffs for reference values [70,73].

**Figure 2:**
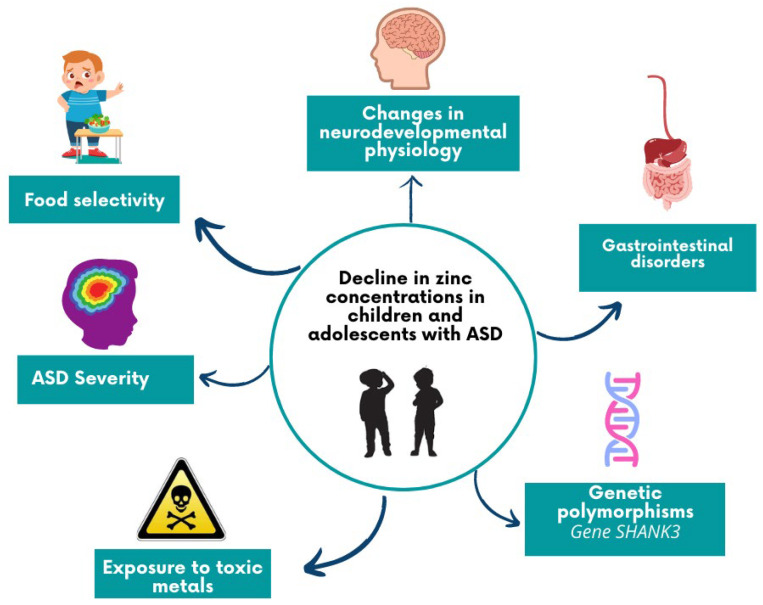
Changes observed with zinc reduction in children and adolescents with ASD.

Importantly, most studies evaluating Zn concentrations in relation to the severity of ASD reported lower concentrations of Zn in serum, hair, and whole blood for individuals with the most serious forms of the disorder [29,44,47,48]. In this regard, the evidence indicates that low concentrations of Zn in individuals with severe ASD forms are related to high concentrations of toxic metals, such as lead (Pb), barium (Ba), mercury (Hg), and lithium (Li) [3,48,74]. Interestingly, the increase in ASD prevalence that has occurred in recent years has been associated with an increased human exposure to environmental toxicity (insecticides, pesticides, toxic metals, etc.) [30], and it has been suggested that high levels of toxic metals and deficiencies of trace elements in children have negative impacts on neurodevelopment [75]. Figure 2 shows the changes observed with zinc reduction in children and adolescents with ASD.

Zn is closely involved in this process, as it is linked to metallothionein (MT), which have a detoxifying role as they bind to other metals, such as copper, cadmium, and lead, which are considered toxic to the human body [76]. These proteins are primarily responsible for the regulation of intracellular Zn homeostasis in the brain [77]. In addition, Zn plays a fundamental role in the nervous system, especially during the neurodevelopmental phase. In this phase, Zn is involved in the regulation of brain morphogenesis through Zinc Finger proteins (Zinc Finger) [11] and in the maturation of oligodendrocytes [78]. Dysfunction in the regulatory roles of Zinc Finger has negative impacts on the development of the nervous system, leading to morphological changes in the brain and neurodevelopmental disorders, including ASD [11]. Low concentrations of Zn have also been associated with changes in brain growth in individuals with ASD [79].

Regarding the underlying mechanisms of Zn functions, low Zn concentrations have been reported to affect activity levels in the postsynaptic region dependent on ProSAP1/Shank2 and ProSAP2/Shank3 [74]. The SHANK3 gene is one of the main genetic components related to ASD [80] and is responsible for the expression of proteins that structure the postsynaptic region of excitatory neurons. In this regard, it has been proposed that Zn supplementation can restore Shank3 levels in the postsynaptic region, modulate excitatory synapses, and consequently improve the ASD phenotype [81].

In addition to the physiological factors of ASD discussed here that directly influence the status of Zn, we must also mention the nutritional issues associated with ASD, which can influence not only the status of Zn but also of other nutrients, thus directly affecting the general nutritional status of these individuals. One of the behavioral characteristics of individuals with ASD that may affect their nutritional status is commonly observed rigid and repetitive eating behavior [82].

On the other hand, food selectivity, when present in individuals with ASD, is generally characterized by a dietary pattern restricted to processed foods, sources of simple carbohydrates, saturated fats, and little dietary fiber [83], thus reducing the supply of animal-derived proteins and essential micronutrients such as Zn. Another relevant factor are the restrictive diets to which some of these individuals are subjected, such as those that exclude gluten and casein [84], thereby decreasing their intake of cereals and dairy products and consequently restricting their supply of calcium, among other nutrients. Although this selective pattern of eating behavior is widely observed in patients with ASD, few studies have evaluated and compared these issues in relation to individuals without the disorder [85]. In this sense, it is important to conduct studies evaluating not only nutrient profiles, but also nutritional status and dietary patterns, which directly influence Zn status, as well as micro-and macronutrients essential to the development of these individuals.

Another important factor that should be considered are the gastrointestinal changes observed in individuals with ASD. In addition to behavioral disorders, gastrointestinal disorders are seen in eight out of ten children [86], with the most frequent symptoms being diarrhea, nausea, vomiting, abdominal pain and distension, and gastroesophageal reflux [87]. Gastrointestinal disorders have a direct impact on Zn status, as it is involved in the mechanisms maintaining the homeostasis of the gastrointestinal tract [88]. In the case of Zn deficiency, fecal excretion decreases, and intestinal absorption to maintain plasma and tissue levels consequently increases; however, this balance is affected only in a severe state of Zn deficiency [89].

The high percentage of included articles (49%) that presented low methodological quality should be considered as a limitation of this study, where confounding factors and selection bias may have influenced the results. We associate the low quality of the studies mainly with the lesser methodological rigor of the participant selection methods, especially with regard to the items of representativeness of the cases and selection of the controls of the NOS scale. An example is the lack of description of the origin of the selected controls, as in the study by Priya and Geeta (2011), and the selection of controls from the same institution where the cases originated, as in the study by Al-Ayadhi (2005) (items 2 and 3 from NOS) [21,29].

However, this review has advantages over reviews previously conducted on the subject, as this is the first systematic review evaluating the status of Zn specifically in children and adolescents with ASD. The lack of delimitation in the evaluation period, language of publication, and the inclusion of studies that evaluated not exclusively Zn, but other trace elements, allowed the inclusion of a considerable number of studies that were conducted in different regions of the world. In addition, data that have not been explored in other reviews were evaluated, such as Zn concentrations in relation to sex, severity of symptoms, and associated conditions, in addition to the biological matrices used for the Zn concentration analyses.

## 5. Conclusions

This review provides evidence that, although it cannot be stated that there is a significant difference in Zn concentrations between individuals with ASD and controls, the reviewed data point to a frequent occurrence of lower concentrations of Zn in individuals with ASD, and that this profile is also possibly related to the severity of the disorder. We emphasize the need for more prospective studies that perform a standardization of the analyses regarding the biological matrix used, as well as greater methodological rigor regarding bias control in the selection of participants.

## Figures and Tables

**Table 1:** Characteristics of the included studies.

Author/ Year	Country	Study Design	Quality	Number of Participants		Average Age		Biological Matrix	Zinc Concentration (ppm)		*p*-Value	Analytical Method
				ASD	Control	ASD	Control		ASD	Control		
Shearer et al., 1982. [16]	USA	Case–Control	3	12	12	8.0 (0.8)	8.4 (0.6)	Hair	175 (73) ^x^	158 (57)	>0.05	ASS
Gentile et al., 1983. [17]	USA	Case–Control	1	47	37	NI		Hair	NI		NI	NI
Wecker et al., 1985. [18]	USA	Case–Control	3	21	12	2–11		Hair	128 (116) ^x^	166 (112)	NI	ASS
Adams et al., 2005. [19]	USA	Case–Control	3	51	40	3–15		Hair	156 AP 163	147	NI	ICP-OES
Laila Y. Al-Ayadhi, 2005. [20]	Saudi Arabia	Case–Control	3	65	80	8.8 (0.5)		Hair	150 (20) ^x^	140 (8)	NI	AAS
Al-Farsi et al., 2013. [21]	Oman	Case–Control	5	27	27	3–14		Hair	5.4 (0.82) ^y^	2.9 (2.2)	0.0001	NI
Tabatadze et al., 2015. [3]	Georgia	Case–Control	3	30	30	4–5		Hair	NI		NI	NI
Skalny et al., 2016. [22]	Russia	Case–Control	5	74	74	5.12 (2.36)	5.11 (2.34)	Hair	124.6 (77.0–174.2)^y^	113.3 (69.4–166.3)	0.365	ICP-MS
Skalny et al., 2016. [23]	Russia	Case–Control	5	33	33	3–8		Hair	130.3 (77.0–175.6) ^y^	101.6 (59.5–149.8)	NI	ICP-OES
Zhai et al., 2019. [24]	China	Case–Control	3	68	58	4.96 (1.01)	4.9 (0.97)	Hair	78.00 ^x^	44.7	<0.001	ICP-MS
Skalny et al., 2020. [25]	Russia	Case–Control	6	109	104	5.18 (1.00)	5.1 (1.05)	Hair	122.3 (86.6–152.9)^y^ AA 110.0 (83.1–139.1)	141.0 (103.9–162.2)	<0.05	ICP-MS
Fiłon et al., 2017. [26]	Poland	Case–Control	2	30	30	5.2 (1.5)	5.09 (1.5)	Hair	99.93 (67.50)^x^	149.66 (42.56)	0.000	NI
Skalny et al. 2017. [27]	Russia	Case–Control	4	70	70	6.4 (2.9)	6.3 (2.9)	Hair/Plasma	122 (77–169)^y^/0.97 (0.89–1.06) ^y^	132 (86–172)/0.98 (0.89–1.05)	0.678 0.617	ICP-DRC-MS
Priya MDL, Geetha A., 2011. [28]	India	Case–Control	4	45	50	4–12		Nail/Hair	c 150.83 (18.09) ^x^ b 192.02 (23.04) ^x^ a 187.44 (22.47)^x^/ c 130.46 (15.65) ^x^ b 172.81 (20.73) ^x^ a 171.92 (20.63) ^x^	193.98 (23.27)/171.68 (20.60)	0.01	AAS
Amen et al., 2020. [29]	Pakistan	Case–Control	5	90	76	9.8 (3.3)		Hair/Urine	NI		NI	ICP-MS
Blaurock-Busch et al., 2011. [30]	Saudi Arabia	Case–Control	5	25	25	6.2 (2.3)	5.2 (1.9)	Hair/Urine	101.042 (52.0)^x^/2213 (1062.20)	149.86 (58.51)/ 890 (450)	0.003 0.32	ICP-MS
Yorbik et al., 2004. [31]	Turkey	Case–Control	3	45	41	6.5 (2.2)	6.7 (2.5)	Plasma/Erythrocytes/Hair	0.0198 (0.0025) ^x^/0.0023 (0.0024)/131,7 (60)	0.0257 (0.0024)/0.003 (0.0029)/184.0 (19)	NI	AAS
Tinkov et al., 2019. [32]	Russia	Case–Control	2	60	30	4.7 (1.8)	4.8 (2.2)	Hair/ Serum	121.59 (77.17–156.1) ^y^/127.45 (92.53–164.5)	152.77 (99.02–179.6)	NI	ICP-MS
Adams et al., 2007. [33]	USA	Case–Control	3	15	11	7.0 (1.7)	6.1 (2.2)	Teeth	100 (20) ^x^	98 (16)	NI	CV-ASS
Wu et al., 2018. [34]	China	Case–Control	5	50	50	2–8		Erythrocytes	Limits of quantification (LOQ) 0.00402	NI	NI	AAS
AJ Russo and Robert de Vito, 2011. [35]	USA	Cohort	4	79	18	11.7 (5.62)		Plasma	78.36 (20.32)^x^	84.42 (24.18)	0.3541	ICP-MS
Qin et al., 2018. [36]	China	Case–Control	5	34	38	4.1 (0.8)	4.2 (1.7)	Plasma	4.30 (1.84) ^x^	5.05 (1.52)	<0.05	ICP-OES
Chehbani et al., 2020. [37]	Tunisia	Case–Control	2	89	70	7.5 (3.0)	7.8 (3.4)	Plasma	0.610 (0.166) ^x^	0.586 (0.179)	0.37	ICP-OES
Deshpande et al., 2019. [38]	India	Case–Control	4	10	10	6–14		Spittle	0.0133 ^x^	0.0274	NI	ICP-OES
Crăciun et al., 2016. [39]	Romania	Case–Control	3	28	28	5.8 (3.1)	5.9 (2.9)	Blood	5.54 (0.78) ^x^	6.14 (0.76)	0.005	ICP-SFMS
Wu et al., 2018. [40]	China	Case–Control	5	113	141	4.9 (2.2)	4.9 (2.1)	Blood	0.122 (0.020) ^x^	0.129 (0.018)	0.05	ICP-MS
Sehgal et al., 2019. [41]	India	Case–Control	6	60	60	3–12 years		Blood	1.04 (0.91–1.18) ^x^	0.90(0.81–1.01)	0.02	ICP-AES
Li et al., 2014. [42]	China	Case–Control	5	60	60	3.78 (1.22)		Serum	0.78 (0.07) ^x^	0.87 (0.08)	<0.001	NI
Skalny et al., 2016. [43]	Russia	Case–Control	3	24	24	6.6 (1.4)	6.5 (0.9)	Serum	1.02 (0.19) ^x^	0.96 (0.12)	0.114	ICP-MS
Min Guo et al., 2018. [44]	China	Case–Control	5	274	97	4.0 (1.1)	4.2 (1.2)	Serum	NI		<000.1	HPLC
Sweetman et al., 2018. [45]	Ireland	Case–Control	5	74	72	2–18		Serum	0.017 (0.002) ^x^	0.017 (0.003)	0.86	ICP-MS
Sultan et al., 2019. [46]	Iraq	Case–Control	2	90	30	2–10		Serum	a 0.022 (0.002) ^x^ b 0.015 (0.001) c 0.014 (0.001)	0.0037 (0.0034)	NI	AAS
Al-Bazzaz et al., 2020. [47]	Jordan	Case–Control	3	35	35	4–12		Serum	0.8434 (0.1828) ^x^	0.9589 (0.1091)	significant at 5%	NI
Hawari et al., 2020. [48]	Syria	Case–Control	4	31	30	3–12		Serum	0.8448 (0.1599) ^x^	0.7997 (0.1372)	NI	NI
Qureshi et al., 2020. [49]	USA	Case–Control	3	21	26	2–5		Urine	5.70 (2.34)	7.44 (3.22)	NI	ICP-OES
Joan Jory and Woody R. McGinnis, 2008.[50]	Canada	Case–Control	3	20	15	3.90 (1.68)	3.87 (1.06)	Erythrocytes	0.2048 (0.0366) ^x^	0.2267 (0.0261)	0.08	ICP-MS
Pakyurek et al., 2018. [51]	USA	Case–Control	4	15	12	5–18		Blood	0.708 (0.1239) ^x^	0.8633 (0.2946)	0.1189	NI
Yasuda et al., 2005. [52]	Japan	Case–Control	3	360	241	0–15		Hair	0.005 (0.0001) ^x^	0.00502 (0.000117)	0.0904	ICP-MS
Elsheshtawy et al., 2010. [53]	Egypt	Case–Control	7	32	32	4.1 (0.8)	4 (0.8)	Hair	304.99 (25.8) ^x^	419.5 (45.96)	0.000	AAS
Semprun-Hernández et al., 2012. [54]	Venezuela	Case–Control	6	30	20	3–17		Serum	1.805 (0.577) ^x^ a 1.904 (0.572) b 1.683 (0.554) c 1.889 (0.667)	2.194 (0.721)	>0.05	AAS
Vergani et al., 2011. [55]	Italy	Case–Control	3	38	32	2–6		Plasma	1.021 (0.100) ^x^	0.808 (0.131)	0.01	ICP-AES
Rezaei et al.,2022. [56]	Iran	Case–Control		44	35	10–11		Urine	0.733	0.764	0.349	ICP-MS
Zhao et al., 2022. [57]	China	Case–Control		30	30	4.2 (1.53)	8 (1.3)	Blood/ Urine	3.8815 (3.319–4.369)/4.354 (1.535–8.069)	4.155 (3.675–4.816)/ 4.833 (1.496–8.487)	0.188 0.918	ICP-MS
Metha et al., 2021. [58]	USA	Case–Control		52	22	2–4		Plasma	0.00075 (0.00016)	0.00088 (0.00035)	0.2309	ICP-MS
Rashaid et a., 2021. [59]	Jordan	Case–Control		50	50	4–12		Hair	185.96 ± 95.35	244.29 ± 183.40	0.038	ICP-AES
Zhang et al., 2021. [60]	China	Case–Control		1342	1293	2–7		Plasma	0.75 (0.64; 0.84) a,b 0.74 (0.66; 0.84) c 0.70 (0.62; 0.82)	0.77 (0.66; 0.87)	0,003	ICP-MS
Skogheim et al., 2021. [61]	Norway	Case–Control		397	1034	NI		Blood	49.66 (48.5–50.85)	52.02 (51.39–52.66)	NI	ICP-SFMS
Zhang J. et al., 2022. [62]	China	Case–Control		30	30	4.03 (1.1)	4.21 (0.9)	Plasma	3.988 (2.995–5.328)	4.279 (3.681–5.306)	0.261	ICP-MS
Jiahui et al., 2021. [63]	China	Case–Control		92	91	2–8		Serum	0.8251 (0.7673–0.9079)	0.8978 (0.8065–0.9489)	0.002	ICP-MS/ICP-AES
Whail et al., 2022. [64]	Malaysia	Case–Control		81	74	3–6		Urine	0.3981 (0.2452)	0.8888 (0.9015)	<0.001	ICP-MS
Gaafar et al., 2021. [65]	Egypt	Case–Control		42	21	3–11		Plasma	0.707 (0.099)	1.14 (0.089)	<0.001	NI
Auda et al., 2021. [66]	Iraq	Case–Control		60	30	3–6		Blood	24.072 (7.359)	33.952 (15.534)	0.0021	EDS

Note: The representation of zinc concentration measurement units were converted and standardized in ppm to facilitate understanding of the data; ^x^ average; ^y^ median; a, mild ASD; b, moderate ASD; c, severe ASD; AA ASD with ADHD; AP ASD with PICA; NI, no information; ICP- OES, Optical Emission Spectrometry with Inductively Coupled Plasma; ICP-MS, Inductively Coupled Plasma Mass Spectrometry; AAS, Atomic Absorption Spectrometry; ICP-AES, Analysis by Spectrometry of Atomic Emission by Induced Plasma; ICP-SFMS, Inductively Coupled Plasma Sector Field Mass Spectrometer; EDS, Energy Dispersive Spectroscopy; ICP-DRC-MS ICP-MS, used in quadrupole-based system equipped with a Dynamic Reaction Cel; HPLC, High Performance Liquid Chromatography; CV-AAS, Cold Vapor Atomic Absorption Spectrometry.

## Data Availability

Not applicable.

## Supplementary Materials

The following are available online at https://www.mdpi.com/article/10.3390/nu15163663/s1. Table S1 presents the search strategy used in each database. Table S2 presents the assessment of methodological quality according to the study type.
