# Supplementary material for: Zinc Status and Autism Spectrum Disorder in Children and Adolescents: A Systematic Review
Source: Nutrients. 2023 Aug 21;15(16):3663. doi: 10.3390/nu15163663 (PMC10459732; doi:10.3390/nu15163663)
Supplement: Supplementary file 1 [file nutrients-15-03663-s001.zip › search estrategy.pdf]

Table S1 search strategy used for each database.

DATA BASE	SEARCH STRATEGY
Pubmed	(zinc OR “trace elements” OR “Trace Element” OR “Element, Trace” OR “Elements, Trace” OR Biometals OR Biometal OR “Trace Minerals” OR “Mineral, Trace” OR “Minerals, Trace” OR “Trace Mineral”) AND (“Autistic Disorder” OR “Disorder, Autistic” OR “Disorders, Autistic” OR “Kanner's Syndrome” OR “Kanner Syndrome” OR “Kanners Syndrome” OR “Autism, Infantile” OR “Infantile Autism” OR Autism OR “Autism, Early Infantile” OR “Early Infantile Autism” OR “Infantile Autism, Early”) AND (child OR children OR adolescent OR adolescence OR pediatric)(zinc OR “trace elements” OR “Trace Element” OR “Element, Trace” OR “Elements, Trace” OR Biometals OR Biometal OR “Trace Minerals” OR “Mineral, Trace” OR “Minerals, Trace” OR “Trace Mineral”) AND (“Autistic Disorder” OR “Disorder, Autistic” OR “Disorders, Autistic” OR “Kanner's Syndrome” OR “Kanner Syndrome” OR “Kanners Syndrome” OR “Autism, Infantile” OR “Infantile Autism” OR Autism OR “Autism, Early Infantile” OR “Early Infantile Autism” OR “Infantile Autism, Early”) AND (child OR children OR adolescent OR adolescence OR pediatric)
Scopus	TITLE-ABS-KEY(zinc OR “trace elements” OR “Trace Element” OR “Element, Trace” OR “Elements, Trace” OR Biometals OR Biometal OR “Trace Minerals” OR “Mineral, Trace” OR “Minerals, Trace” OR “Trace Mineral”) AND TITLE-ABS-KEY(“Autistic Disorder” OR “Disorder, Autistic” OR “Disorders, Autistic” OR “Kanner's Syndrome” OR “Kanner Syndrome” OR “Kanners Syndrome” OR “Autism, Infantile” OR “Infantile Autism” OR Autism OR “Autism, Early Infantile” OR “Early Infantile Autism” OR “Infantile Autism, Early”) AND TITLE-ABS-KEY(child OR children OR adolescent OR adolescence OR pediatric)
Lillacs	(criança OR adolescente OR adolescência) AND (zinco OR oligoelemento OR “Elementos Traço” OR “Minerais-Traço” OR “Mineral-Traço” OR Biometal) AND (Autismo OR “Autismo Infantil” OR “Síndrome de Kanner”)
