# Supplementary material for: Zinc Status and Autism Spectrum Disorder in Children and Adolescents: A Systematic Review
Source: Nutrients. 2023 Aug 21;15(16):3663. doi: 10.3390/nu15163663 (PMC10459732; doi:10.3390/nu15163663)
Supplement: Supplementary file 1 [file nutrients-15-03663-s001.zip › Table S2-updated.pdf]

Table S2. Methodological quality according to study type.

Cohort—Newcastle-Ottawa Scale

	**Seletion**				**Comparability**	**Outcome**			**Total**
Quality criteria	1. Representativeness of the exposed cohort	2. Selection of the non-exposed cohort	3. Ascertainment of exposure	4. Demonstration that outcome of inrerest was not present at start of study	1. Comparability of cohorts on thebasis of the design or analysis	1. Assessment of outcome	2. Was follow-up long enough for outcomes to occur?	3. Adequacy of follow up of cohorts	
[35]	*				*	*	*		4

Case - control—Newcastle–Ottawa Scale

	**Seletion**				**Comparability**	**Exposure**			
Quality criteria	1. Is the case definition adequate?	2. Representativeness of the cases	3. Selection of Controls	4. Definition of Controls	1. Comparability of cases and controls on the basis of the design or analysis	1. Ascertainment of exposure	2. Same method of ascertainment for cases and controls	3. Non-Response rate	
[16]			*				*	*	3
[17]							*		1
[18]			*		*		*		3
[19]			*		*		*		3
[20]	*					*	*		3
[21]	*			*		*	*	*	5
[3]					*		*	*	3
[22]	*				*	*	*	*	5
[23]	*					*	*		3
[24]	*					*	*		3
[25]	*			*	**	*	*	*	7
[26]				*			*		2
[27]	*				*	*	*		4
[28]	*				*		*	*	4
[29]	*	*	*			*	*		5
[30]	*		*	*			*	*	5
[31]	*			*		*			3
[32]	*						*		2
[33]			*	*			*		3

[34]	*	*	*		*	*		5
[36]	*	*	*		*	*		5
[37]	*			*				2
[38]	*		*			*	*	4
[39]	*					*	*	3
[40]	*			*	*	*		4
[41]	*	*		*	*	*	*	6
[42]	*	*	*			*	*	5
[43]	*	*	*		*	*		5
[44]	*			*	*	*		4
[45]	*		*	*	*	*		5
[46]				*		*		2
[47]	*				*	*		3
[48]	*			*	*	*		4
[49]	*	*				*		3
[50]	*		*			*		3
[51].	*		*		*	*	*	4
